# In the room but not on the byline: trust, fear, and the human role in addiction science in the age of AI

**DOI:** 10.3389/fsoc.2026.1786377

**Published:** 2026-03-03

**Authors:** Anees Bahji

**Affiliations:** 1 Department of Psychiatry, University of Calgary, Calgary, AB, Canada; 2 Department of Community Health Sciences, University of Calgary, Calgary, AB, Canada; 3 Hotchkiss Brain Institute, University of Calgary, Calgary, AB, Canada

**Keywords:** artificial intelligence, idea generation, research, research writing, scientific process

## Introduction

I write this as an addiction psychiatrist, a clinician-scientist, a PhD candidate, and a deputy editor of the Canadian Journal of Addiction. I encounter artificial intelligence not as a hypothetical future, but as a recurring practical problem-one that surfaces in manuscripts, reviewer comments, editorial meetings, and disclosure forms with increasing regularity. Questions about AI are no longer about whether it will affect scientific publishing, but about what it is already revealing about how science actually works.

At two o'clock in the morning, with a manuscript deadline looming after a full clinical day, I have used ChatGPT to rephrase a stubborn paragraph. I have used it to check reference formatting. I have relied on AI-powered tools to screen thousands of abstracts for systematic reviews and to summarize dense policy documents that would otherwise take hours. None of this felt unethical. None of it felt like cheating. It felt efficient. It felt pragmatic. It felt human. By human, I do not mean that the technology itself possesses agency or intention, but that its use reflects human judgment operating under real-world constraints-fatigue, time pressure, and responsibility for the final product.

Yet when submitting manuscripts, I am increasingly asked to disclose this use as though it were a conflict of interest. AI cannot be listed as an author. It cannot be cited. It cannot take responsibility. And yet it is clearly present throughout the research workflow. AI is not on the byline-but it is very much in the room.

What drives many of these policies is not only concern about accuracy or misconduct, but something more affective: fear, and a corresponding lack of trust. The scientific enterprise rests on assumptions we rarely state explicitly. We assume researchers act in good faith. We assume the data are real. We assume the analyses were actually conducted. We assume the methods reflect what was done. We assume the writing faithfully represents the work rather than obscuring it. Peer review depends on these assumptions. Without them, the system collapses.

AI did not create these vulnerabilities. It exposed them.

Our response to that exposure has been strikingly asymmetric. Using AI to fabricate data, falsify results, or invent citations is rightly condemned as scientific misconduct. But using AI to refine language, structure, or clarity is often treated with suspicion, as though linguistic polish itself undermines integrity. Writing is implicitly framed as secondary to “real” research, despite the fact that knowledge generation, synthesis, and translation are inseparable in practice. Research that is never clearly communicated might as well not exist.

Recognizing writing as constitutive of knowledge production does not require assigning epistemic agency to every tool involved in that process. Scientific authorship has always relied on assistance-from statisticians, editors, translators, and software-without dissolving responsibility or authorship. The ethical distinction does not hinge on whether writing matters, but on who retains judgment over truth claims. AI-assisted refinement alters form, not ownership of meaning; fabrication, by contrast, substitutes generated content for accountable human judgment.

This opinion advances a simple but necessary claim: AI use in research is not inherently a replacement for human scientific judgment-it is, in most legitimate cases, a refinement of it. The ethical failure is not using AI to improve clarity, efficiency, or accessibility. The ethical failure is using AI to fabricate, falsify, or evade responsibility.

## Fear of replacement and what it clarifies

I will admit something less comfortable. Part of the anxiety around AI is personal. I am occasionally afraid that AI might replace aspects of my own role. In some narrow domains, it already performs certain tasks faster than I do: summarizing literature, drafting text, synthesizing arguments, generating outlines. In those moments, the question becomes unsettlingly direct: If AI can do this, what am I for?

That fear turns out to be clarifying rather than paralyzing. It forces a more honest accounting of what is actually unique about human scientific contribution. My value is not typing speed. It is not flawless grammar. It is not even synthesis in the abstract. It lies in judgment: deciding what matters, what is plausible, what is ethical, what is clinically meaningful, and what responsibility I am willing to publicly stand behind.

AI can generate text. It cannot own consequences.

## Refinement is not deception

Influencers routinely use AI to refine social media posts-adjusting tone, clarity, and reach. Few would argue this constitutes deception. In academic writing, using AI to refine language performs an analogous function. It does not alter the underlying data, analyses, or interpretations. It makes the work more legible.

In addiction science, this matters deeply. Our field sits at the intersection of medicine, policy, lived experience, and public discourse. Poorly written science does not merely inconvenience readers; it distorts meaning, limits uptake, and reinforces inequities-particularly for non-native English speakers. Treating clarity as ethically suspect while tolerating opaque prose is a strange inversion of scientific values.

Using AI to fabricate data is misconduct. Using AI to refine language is not. Conflating the two erodes trust rather than protecting it.

## What AI has actually exposed about science

The unease surrounding AI reflects an uncomfortable truth: much of scientific publishing already operates on trust rather than verification. Reviewers rarely re-run analyses. Editors cannot audit raw data for every submission. We rely on professional norms and accountability.

AI did not make science fragile; it revealed where it already was.

Hallucinations are a genuine risk, but not merely a technical one. They are an epistemic threat precisely because fluent prose can masquerade as knowledge. When linguistic confidence substitutes for evidentiary accuracy, peer review risks becoming stylistic proofreading rather than scientific evaluation. The danger lies not in refinement, but in unexamined authority.

AI has exposed a longstanding vulnerability in science: our reliance on coherence as a proxy for epistemic authority. A recent and tragic case in which an individual died by suicide after interacting with an AI chatbot has intensified public concern about large language models. It is tempting to frame such cases as evidence that AI itself is harmful or malevolent. I find that framing unhelpful. A more uncomfortable interpretation is that AI did exactly what it was designed to do: mirror user input, align with conversational cues, and generate responses that feel coherent and validating. It did not generate suicidal intent, nor did it possess the capacity to recognize when validation becomes containment failure.

In psychiatry, we are trained to understand that empathy without judgment-and reflection without boundaries-can be dangerous. AI has not created this ethical problem; it has exposed what happens when systems designed for linguistic refinement are implicitly entrusted with moral or clinical authority they do not possess. The failure was not one of refinement, but of misplaced responsibility. Authority derived from coherence rather than accountability is already a structural risk-one that AI makes more visible and more scalable.

Scale amplifies this risk. Humans can fabricate papers; AI allows fabrication at industrial volume. Paper mills, fake peer review, and mass-generated manuscripts are already emerging realities. Blanket prohibition is a poor response. Detection tools are unreliable and disproportionately flag non-native English writers. Fear-driven policies push AI use underground and discourage honesty.

The solution is not reflexive fear, but institutional and epistemic literacy. Literacy here means more than technical familiarity with AI systems. It requires understanding where AI is appropriate, where it is not, and which responsibilities remain irreducibly human.

## Peer review, burden, and responsibility

Peer review in addiction science is already strained. Clinician-reviewers juggle heavy caseloads, administrative demands, and academic expectations. AI-assisted writing accelerates submission volume without expanding reviewer capacity.

Here again, refinement matters. AI can assist with statistical checks, reference verification, and image analysis. It can reduce cognitive load without replacing judgment. What it cannot do-and must not be allowed to do-is assume responsibility for evaluative decisions.

Uploading manuscripts to public large language models for “review assistance” violates confidentiality. Using AI to support internal checks does not. The difference is not technological; it is ethical.

## Disclosure without stigma

Current disclosure practices lack nuance. What counts as meaningful AI use? Spellcheck? Paragraph rewriting? Abstract screening? Without clarity, disclosure becomes performative rather than informative-and begins to resemble a moral test rather than a transparency mechanism.

A functional framework would ask:

- What task was AI used for?
- Did it alter content or only form?
- Could the same task reasonably have been performed by a human assistant?

Disclosure should build trust, not punish efficiency or honesty.

## Conclusion: what remains human

AI is not replacing science. It is refining how science is written, processed, and shared. The real threat lies not in responsible AI use, but in our reluctance to confront what science has always required: trust, accountability, and judgment.

AI can scale information. It can generate text, summarize evidence, and surface patterns at speeds no human can match. What it cannot do—at least for now—is what science ultimately demands of its practitioners. It cannot weigh competing values. It cannot situate findings within lived human contexts. It cannot decide when uncertainty warrants caution, when evidence is sufficient for action, or when silence would be more ethical than publication. It cannot be held morally or professionally accountable for the consequences of its outputs.

My role—as a clinician, a scientist, and an editor—is not simply to produce information. It is to interpret it, to contextualize it, to judge its relevance and limits, and to stand publicly behind those judgments. It is to bring not just technical competence, but ethical responsibility and human concern to decisions that affect patients, policy, and public trust. These are not inefficiencies to be automated away; they are the core of scientific and clinical work.

If AI has clarified anything, it is this: the value of human contribution in science was never primarily linguistic or mechanical. It has always resided in judgment, interpretation, and accountability—in the willingness to say *this matters, this is uncertain, this may cause harm*, and *I am responsible for this claim*. AI can assist that work. It cannot replace it.

That responsibility remains human work.

